# The First Report on Transgenic Hairy Root Induction from the Stem of Tung Tree (*Vernicia fordii*)

**DOI:** 10.3390/plants11101315

**Published:** 2022-05-16

**Authors:** Hongyu Jia, Junjie Chen, Lin Zhang, Lingling Zhang

**Affiliations:** 1 Key Laboratory of Cultivation and Protection for Non-Wood Forest Trees, Ministry of Education, College of Forestry, Central South University of Forestry and Technology, Changsha 410000, China; jiahongyu970926@163.com (H.J.); 19862918526@163.com (J.C.); 2 CAS Key Laboratory of Plant Germplasm Enhancement and Specialty Agriculture, Wuhan Botanical Garden, The Innovative Academy of Seed Design, Chinese Academy of Sciences, Wuhan 430074, China; 3 Center of Economic Botany, Core Botanical Gardens, Chinese Academy of Sciences, Wuhan 430074, China

**Keywords:** *Vernicia fordii*, oil-bearing woody plant, *Agrobacterium rhizogenes*, hairy roots, Euphorbiaceae

## Abstract

Tung tree (*Vernicia fordii*) is an industrially important oil-bearing woody plant of the Euphorbiaceae family. Functional studies of tung tree at the molecular level are limited by the lack of an efficient transgenic system. The *Agrobacterium rhizogenes*-mediated hairy root generation system is an alternative to typical plant transformation systems. However, its application in many plants has been blocked due to the inability of existing methods to induce hairy roots. Thus, it is critical to build a method suitable for the hairy induction of the specific plant of interest. In this study, a modified method for tung tree was developed, and it is the first report that hairy roots could be effectively induced in the stem of tung tree. With the method, an average of 10.7 hairy roots per seedling were generated in tung tree, approximately 67% of seedlings produced transgenic hairy roots and approximately 13.96% of the hairy roots of these seedlings were transgenic. This modified method is also suitable for the hairy root induction of two other oil-bearing plants of the Euphorbiaceae family, *Ricinus communis* and *Vernicia montana*. This modified method will accelerate functional studies of tung tree at the molecular level and also shed light on plants lacking a transgenic system.

## 1. Introduction

Tung tree (*Vernicia fordii*) is an oil-bearing woody plant species in the Euphorbiaceae family. The oil extracted from tung tree seeds, called tung oil, is an excellent dry oil and resistant to heat and cold, acid and alkali and corrosion and has insulating properties. Thus, it widely used in paints and varnishes and in products that require polymerization. As a good-quality paint, tung oil is often brushed on the bottom of sea vessels to reduce the adhesion of seaweed and other substances [1]. In addition, tung oil is also used in the electronic industry, for instance, for the production of circuit boards [2]. In addition, there are many other products with tung oil as a raw material, such as inks, cosmetics and pharmaceuticals [3,4]. Thus, tung tree is an economically important woody oil plant.

The unique value of tung oil is largely due to the unusual trienoic fatty acid (FA) α-eleostearic (α-ESA, 18:3Δ^9*cis*,11*trans*,13*trans*^), which comprises approximately 80% of the FA component in the plant and confers superb drying properties to the oil [5].

Tung tree has been cultivated in China for over one thousand years [6]. It is mainly distributed in Hubei, Guizhou, Sichuan, and Hunan provinces, and the Chongqing municipality [7]. In history, tung oil was once China’s bulk export commodity for foreign exchange. During this period, many local varieties were developed, including the well-known “Jinsi” tung tree of Hubei province [8]. However, this golden era ended when competitive substitutes for tung oil products appeared in the late 1980s [9]. Consequently, this resulted in the sharp decrease in tung oil price and large-scale cutting of tung trees. Since then, tung trees have had a semi-wild status, and lots of local varieties basically disappeared due to cutting or natural death. Recently, the tung tree has received increasing attention as tung oil has been revealed as an excellent environmentally friendly feedstock in comparison to its substitutes.

The tung tree genome has been published by two research groups: Cui et al. [10] and Zhang et al. [11]. These studies have provided valuable genomic data for molecular breeding of tung tree, and since then, there has been a considerable amount of effort in the molecular breeding of this species [12,13,14,15,16,17,18]. However, these efforts have been hampered by the lack of an efficient genetic transformation system.

The Agrobacterium-mediated plant transformation system has been widely used in plant genetic engineering in recent decades. It has, however, been proven difficult to apply the system to many unique medicinal or woody plants, notably the tung tree and peach [19]. *Agrobacterium rhizogenes (Rhizobium rhizogenes*), a relative of *Agrobacterium tumefaciens*, could induce hairy roots (adventitious roots) upon wounding and infection of plant leaves or stems. Target genes can be transferred and incorporated in the host plant genome by infection by *Rhizobium rhizogenes*, and thus a certain percentage of transgenic roots could be obtained by this technology. This system has been extensively applied to numerous plant species in a variety of biotechnological studies, such as production phytochemical compounds [17,20,21,22] and confirmation of gene function [23,24]. Meng et al. [23] developed an efficient root transformation system using *Rhizobium rhyzogenes* that was successful for many medicinal and woody plant species, but it did not induce hairy roots for several plants selected in their study, such as castor (*Ricinus communis*), a member of the Euphorbiaceae family [23]. Likewise, we were unable to use this method with tung tree, which of interest to us. As noted, the tung tree is also in the Euphorbiaceae family, and perhaps this method is not as widely effective in this family as it appears to be in others. Therefore, it is critical to develop a new or modified *Rhizobium rhizogenes*-mediated root transformation method for tung tree.

In this study, we developed a hairy root induction method based on the method of Meng et al. [23] and hairy roots were successfully induced from the stems of tung tree seedlings. It is the first time that we have built a method for accomplishing this. Using the modified method, we also successfully induced hairy roots in castor and another industrial oil-producing plant in Euphorbiaceae, *Vernicia montana.* Further, it was found that approximately 67% of the tung tree seedlings treated with this method harbored transgenic roots, and 13.96% of the hairy roots generated in these seedlings were transgenic. We propose that this modified method provides a new tool for studying the genetics of the tung tree and will, in the near future, greatly enhance our understanding of this important species, as well as related species.

## 2. Results

### 2.1. Hairy Root Induction in the Stem of Tung Tree

**Figure 1:**
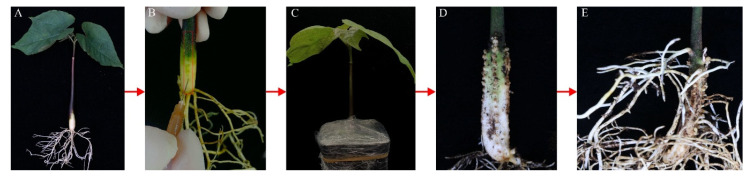
The flow chart of the modified K599-mediated hairy root induction method used for tung tree. (**A**) Photograph of a tung tree seedling 14 days after germination, before inoculation. (**B**) The stem segments as prepared for injection and the method of injection are shown. Inoculum of K599, which had a measured OD_600_ approximately equal to 0.2, was injected along the four equally spaced lines in the 8 cm stem segment above the root. The four lines are distributed in four opposite directions, with equal spacing and an angle of 90° between the lines. (**C**) The surface of the mud of the pot was covered with a transparent plastic wrap after injection. (**D**) Calli that developed two weeks after injection along the injection lines are shown. (**E**) Stem segments four weeks after injection, showing the hairy roots that were produced along the injection lines.

Our goal was to develop a method for the transformation of tung tree based on *Rhizobium rhizogenes* induction of hairy roots in stems of these plants. We modified the method of Meng et al. [23] to develop a root transformation method that would be effective in tung tree. These modifications are described in the Section 4, but to summarize, there were two major changes to the original method. First, we expanded the injection site, from a 0.5 cm region of stem to a whole 8 cm stem region above the roots, as shown in Figure 1A. This expanded injection site was then injected with the prepared inoculum (Figure 1B). Second, a humid environment at the injection site would support the generation of hairy roots. This inspired us to submerge the injected stem in mud and cover the surface of the mud in the pot with a transparent plastic wrap, leaving no gap between the stem and plastic wrap (Figure 1C). As shown in Figure 1D, many punctate calli developed along the injection lines two weeks after the injection, and many hairy roots were induced four weeks after injection (Figure 1E).

### 2.2. The Applicability of the Modified Method to Two Other Industrially Important Members of the Euphorbiaceae Family

Two other members of the Euphorbiaceae family also have high levels of unusual FAs that are valued by industry. Fatty acids of castor and *V. montana* seeds are about 90% ricinoleic acid and 70% α-eleostearic acid, respectively. Hairy roots have not been successfully induced in castor by the Meng et al. method [23]. Given the success of our modified method with tung tree, we wondered if it would also be applicable to these two related species.

**Figure 2:**
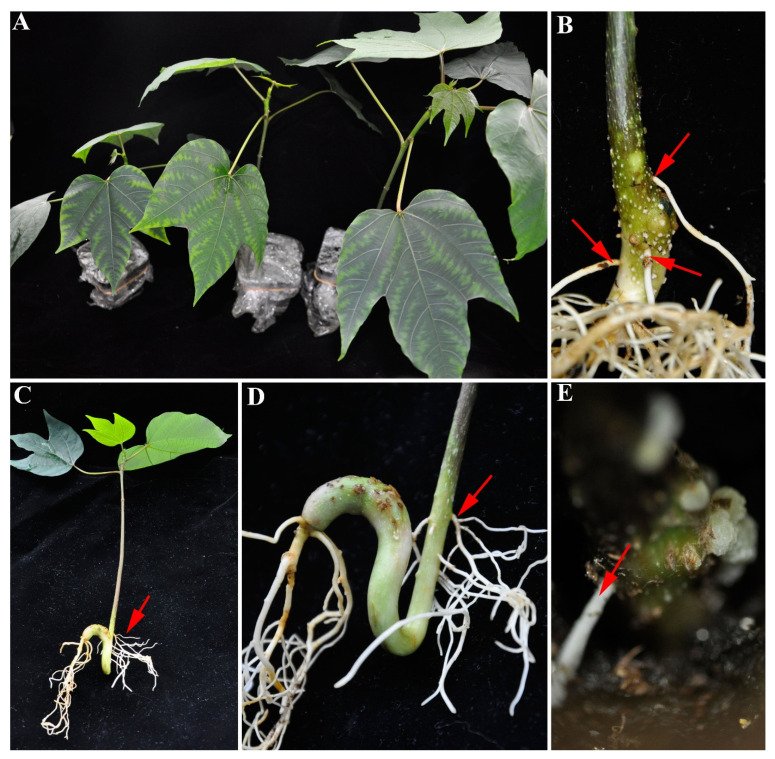
Induction of hairy roots in the stem of *Vernicia montana* by the modified method shown in Figure 1. (**A**) Seedlings after injection are shown. (**B**) Stems four weeks after injection. Hairy roots and many tiny calli are apparent. (**C**) The whole seedling, with hairy roots, four weeks after injection. (**D**) The stem of seedlings from “C” that had hairy roots but no calli. (**E**) Stems of seedling four weeks after injection, showing callus and hairy roots. The arrows indicate the induced hairy roots.

**Figure 3:**
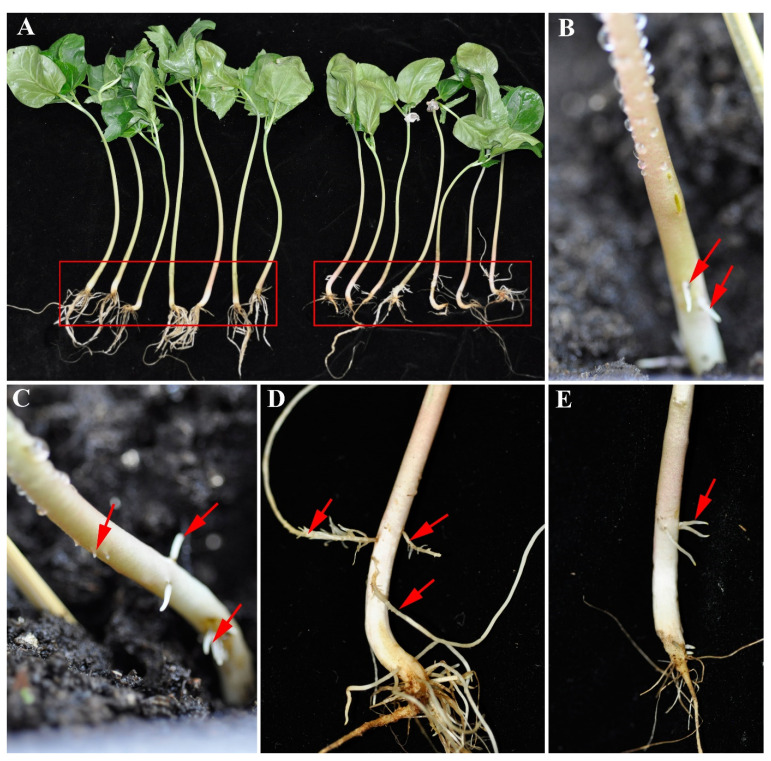
Induction of hairy roots in the stem of castor by the modified method shown in Figure 1. (**A**) Castor seedlings with or without hairy roots 4 weeks after injection. (The red boxes indicate the stem region). (**B**–**E**) Hairy roots that formed on the stem of different seedlings of castor, as a result of inoculation. All the hairy roots arose directly from the epidermis of the stem. Few calli were observed. The arrows indicate the induced hairy roots.

The results are shown in Figure 2 for *Vernicia montana* and in Figure 3 for castor. Using the modified method, hairy roots were successfully produced in the stems of both. In *Vernicia montana*, we also observed local enlargement (Figure 2B) and bulk callus (Figure 2E) in the injection region; this is similar to our results with tung tree. For castor, there was little local enlargement and few calli (Figure 3A,B) in all the test seedlings, and hairy roots arose directly from the epidermis of the stem (Figure 3C–E). We noted that four weeks after injection, of the three plants, only the castor seedlings with hairy roots were obviously shorter than those with no hairy roots (Figure 3A). Castor is a herbal plant, while both tung tree and *Vernicia montana* are woody plants, which may lead to the different phenotypes during the process of hairy root induction by this modified method.

### 2.3. Transgenic Hairy Root Induction in the Stem of Tung Tree

**Figure 4:**
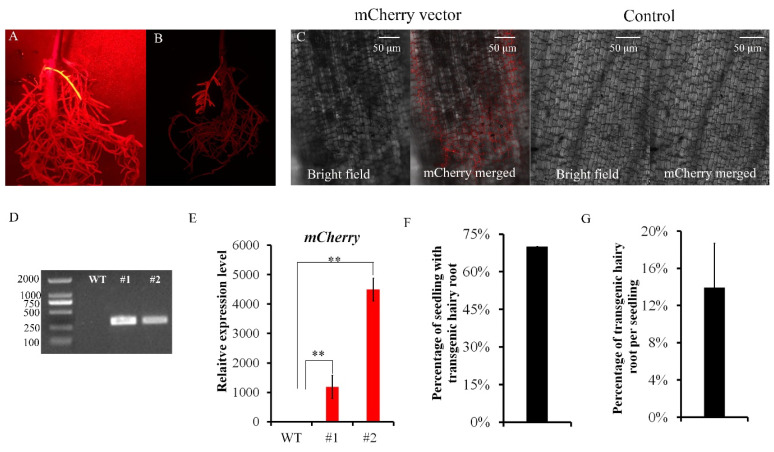
Induction and identification of transgenic hairy roots in the stem of tung tree seedling. (**A**,**B**) The hairy roots six weeks after injection. Red fluorescence is apparent in transgenic roots. (**C**) Signal for the red fluorescence protein in transgenic hairy roots, as determined by a confocal laser-scanning microscope (excitation wavelength is 633 nm). (**D**) The amplification bands of mCherry genes (coding the DsRed fluorescence protein) based on genomic DNA of transgenic hairy roots and wild-type (WT) roots. (**E**) The relative expression level of mCherry gene in the transgenic hairy roots and WT roots by qRT-PCR amplification. The data present the average of three repeats. ** Two-tailed Student’s t test, *p* < 0.001. (**F**)**.** The average fraction of the seedlings producing the transgenic hairy roots. (**G**) The average fraction of the transgenic hairy roots per seedling.

Six weeks after the injection with the prepared inoculum containing empty vector pSAK277-RFP, transformed roots were identified with a hand-held LUYOR-3415RG and a double fluorescent protein observation. Coding #1 and #2 are the transgenic hairy roots of tung tree. The transgenic hairy roots (#1, #2) can be distinguished from the wild-type (WT) roots by their red color in this light (Figure 4A) or because they are obviously brighter than the surrounding WT roots (Figure 4B). The signal of the double fluorescent protein observation in transgenic hairy roots was further confirmed with a confocal laser-scanning microscope (Zeiss; LSM710, excitation wavelength is 633 nm) (Figure 4C), with the WT root as control.

We performed two PCR analyses, one based on genomic DNA (Figure 4D) and another qRT-PCR amplification based on cDNA (Figure 4E). From the results of both, we concluded that the mCherry gene was transferred into the tung tree genome and expressed in the hairy roots. From these results, we also concluded that our method of the *Rhizobium rhizogenes*-mediated root transformation system was successful in the tung tree.

Thirty days after injection, the number of plants producing hairy roots was counted among the injected plants. Then, the ratio of the number of plants producing hairy roots to the total number of injected plants was calculated. The number of transgenic hairy roots was counted and the ratio of the number of transgenic hairy roots to the total number of hairy roots was also calculated. The number of hairy roots induced in each seedling varied from 0 to 24, with an average of 10.71 hairy roots per seedling. The number of transgenic hairy roots per seedling varied from 0 to 6, with an average of 2.29 transgenic hairy roots per seedling. The average fraction of the seedlings producing transgenic hairy roots was 67% (Figure 4F), and approximately 13.96% of the hairy roots produced by these seedlings were transgenic (Figure 4G). From these data, we concluded that the modified method efficiently induced transgenic hairy roots in the stem of tung tree.

## 3. Discussion

### 3.1. Transgenic Hairy Root Induction Is a Feasible Way to Develop a Genetic Transformation System for Many Plants Lacking a Reliable Transgenic System

A reliable transgenic system is critical for the investigation of molecular functions of genes of interest. However, it is quite difficult to develop a stable transgenic system for many economically important woody plants. Due to this lack, less is known about these plants at the molecular level than for species more amenable to transformation. Usually, genes of interest in the absence of a transgenic subject must be functionally analyzed in a heterogenic plant transfer system. However, the original function of many genes often cannot be accurately verified under the heterogenic system.

To solve the above problem, an alternative method, the transgenic hairy root induction technique based on *Agrobacterium rhizogenes*, is constructed and widely used in many plants [16,17,18,19,20,21,22,23,24,25,26,27,28,29,30]. Several strains of *Agrobacterium rhizogenes*, such as *Agrobacterium rhizogenes* LBA9402 [26], *Agrobacterium rhizogenes* A4 [27] and *Rhizogenes rhyzogenes* K599 [28], were reported to be used for hairy root induction. Xu et al. [26] built a transgenic hairy root induction system in spinach by infecting its explant stems with *Agrobacterium rhizogenes* strain LBA9402. Zhao et al. [29] also utilized *Agrobacterium rhizogenes* LBA9402, and established a transformation system for hairy root production of the alfalfa variety WL-525HQ. Zhang. [27] used direct injection of *Agrobacterium rhizogenes* A4 into the stem of *Chenopodium quinoa*, and successfully induced hairy roots. Yang. [28] established a genetic transformation system for hairy roots by injecting *Rhizogenes rhyzogenes* strain K599 into the hypocotyl cotyledon nodes of *Caragana intermedia*. Xie et al. [30] infected seedlings of *Carya illinoinensis* “Zhong Shan” with *Rhizogenes rhyzogenes* strain K599, and also established a transformation system of *Carya illinoinensis*. Additionally, Meng et al. [23]. developed a hairy root method with *Rhizogenes rhyzogenes* strain K599 that could be suitable for 12 planted species, including varieties of herbaceous and woody plants. Taken together, transgenic hairy root induction is a feasible way to obtain transgenic materials for many plants lacking a reliable transgenic system.

### 3.2. The Critical Factors in the Hairy Root Induction of Plant Seedlings

It is quite difficult to obtain transgenic tung tree seedlings by establishing a stable genetic transformation system. To date, there are no studies on stable genetic transformation systems in tung tree. This lack of a transgenic system has indeed severely hampered the study of functional genes of tung tree. Therefore, the establishment of a genetic transformation system is one of the most pressing challenges in research work on tung tree.

As discussed in Section 3.1, a transgenic hairy root induction system has been constructed in many plants. However, we are unaware of any transgenic system for tung tree at present. Of these reports on the transgenic hairy root systems, Meng et al. [23] developed a simple and efficient method for transgenic hairy roots, and this method is applicable to a wide range of plant species, including woody plants, medicinal plants and fruit trees. However, it was not effective in some plants, such as castor selected in their study. We also could not induce hairy roots in the tung tree of interest here based on this method.

In this study, we modified the Meng et al. method [23] and developed a simple and effective technique to obtain adventitious roots by using *Rhizobium rhizogenes* strain K599 to infect living seedlings of tung tree, castor and *Vernicia montana*, three kinds of oil-bearing plant in the Euphorbiaceae family. Compared to the Meng et al. method [18], three aspects were altered in this modified method. We applied the prepared inoculum at a lower concentration (OD_600,_ 0.2) than in the original method (OD_600,_ 0.3–0.4). We also used a larger injection site on the stem than they used (8 cm above the roots, compared to 0.5 cm in the original method), as shown in Figure 1B. Finally, we submerged the entire stem after injection into mud and covered it with transparent plastic wrap to maintain a humid environment instead of being exposed to the air. The inoculum concentration, injection area and humid soil environment should contribute to the successful induction of hairy roots, probably by increasing the occurrence of hairy roots. Thus, these alterations should cause the successful induction of hairy roots of castor (Figure 3) and tung tree (Figure 1), which was not possible with the original Meng et al. method. Thus, this modified method based on the Meng et al. method [23] may be suitable for a wide range of other plants.

### 3.3. Significance of Transgenic Hairy Roots Induced from the Stem in the Study of Tung Tree

With the successful construction of the transgenic root induction method, the function of many genes could be verified in the native plants lacking a steady genetic transformation system. For instance, obtaining relevant transgenic root materials of alfalfa accelerated the study of the aluminum tolerance gene, providing an effective tool of functional gene research in alfalfa [29]. A transgenic hairy root system of *Carya illinoinensis* laid the foundation for breeding new varieties of *Carya illinoinensis* by improving agronomic traits [30]. In this study, the transformation efficiency of transgenic roots in tung tree is relatively high: approximately 67% of the tung tree seedlings produced transgenic hairy roots, and of these roots, approximately 13.96% were transgenic. This showed that an effective transgenic root method was developed for tung tree. With the method, we could directly carry out the investigation of molecular regulation of interesting transcription factors in the tung tree. In addition, this development of transgenic root systems also allows gene function studies by overexpression and silencing techniques or gene editing tools such as CRISPR/Cas9. Generally speaking, this technique can provide a convenient platform for the mining and identification of functional genes in tung tree, and could benefit the rapid development of molecular breeding in tung tree in the future. In addition, this method will provide a technical reference for other plants for which it is difficult to establish genetic transformation systems.

## 4. Materials and Methods

### 4.1. Plant Materials

Seeds of tung tree (*Vernicia fordii),* castor (*Ricinus communis* L.) and *Vernicia montana* were sown in pots (6.5 × 6.5 cm) containing pindstrup substrate. A single seed was planted in each pot. The pots were transferred to a greenhouse at a constant 24 °C with a 16 h light/8 h dark cycle. The plants were grown in the greenhouse for 14–21 days after germination, when the seedlings, shown in Figure 1A, were used in the experiment.

### 4.2. Preparation of Inoculum for Transformation

*Rhizobium rhizogenes* strain K599 [23], obtained from Beijing Forest University, was used to transform the seedlings of tung tree. A fresh single colony of K599 was cultured in 5 mL of LB liquid medium containing 20 mg/L rifampicin and shaken at 200 rpm at 28 °C overnight, which was used to develop the method suitable for the hairy root induction of tung tree. Additionally, a fresh single colony of strain K599 harboring the empty vector pSAK277-RFP was cultured in 5 mL of LB liquid medium containing 20 mg/L rifampicin and 50 mg/L kanamycin, and shaken at 200 rpm at 28 °C overnight, which was used to test the transformation efficiency of the modified method. The pSAK277-RFP, derived from pSAK277, contained a reporter system of a monomeric derivative of DsRed fluorescent protein coding by the mCherry gene [25], which facilitated the rapid identification of transgenic roots in this study. The next morning, 1 mL of the bacterial liquid from the two kinds of cultures was added to 50 mL LB liquid medium containing the same antibiotics as the 5 mL LB mentioned before, respectively, and shaken at 200 rpm at 28 °C until the OD_600_ reached 0.6-0.8. At this time, the cultures were centrifuged at 6000 rpm for 10 min at room temperature, washed with MES buffer (10 mM MES, 10 mM MgCl_2_, 0.1 mM acetosyringone and 0.5% glucose) and re-centrifuged as before. The cultures were resuspended in 2 mL MES buffer. The two kinds of cultures were diluted to an OD_600_ approximately equal to 0.2 with MES buffer in preparation for inoculation into the plant by injection.

### 4.3. Hairy Root Induction and Confirmation of Positive Hairy Roots

We used stem segments, 8 cm in length, for injection. Four equally spaced lines were chosen on the 8 cm segments in the stem above the roots (see the flow chart in Figure 1). The four lines were distributed in four opposite directions, with equal spacing and an angle of 90° between the lines. The prepared inoculum was injected, beginning at the bottom of the stem segment, along each of the four lines to the position 8 cm above. Stem segments were observed every two weeks after injection. After six weeks, the number of hairy roots per plant was counted and transgenic hairy roots were identified with a hand-held double fluorescent protein observation tool (LUYOR-3415RG) and a pair of specific glasses with LUV-50A. Later, the RNAs of the roots with red fluorescence and the wild hairy roots without red fluorescence were extracted with an RNA extraction kit (BioTeck Corporation, Wuxi, China). One microgram of the total RNA extracted was reverse transcribed to cDNA using the PrimeScript™RT Kit (Cat. RR047A, TaKaRa, Shiga, Japan). The primers used for PCR amplification, which were based on genomic DNA, and qRT-PCR amplification, based on cDNA, are listed in Table S1. We calculated the fraction (%) of seedlings with transgenic roots (the ratio of the number of plants producing transgenic hairy roots to the total number of injected plants) and the fraction (%) of these roots that were transgenic (the ratio of the number of transgenic hairy roots to the total number of hairy roots).

## 5. Conclusions

In this study, a modified method for tung tree was developed, and it is the first report that hairy roots could be effectively induced in the stem of tung tree. With the method, an average of 10.7 hairy roots per seedling were generated in tung tree, approximately 67% of seedlings produced transgenic hairy roots and approximately 13.96% of the hairy roots of these seedlings were transgenic. This modified method is also suitable for the hairy root induction of two other oil-bearing plants of the Euphorbiaceae family, *Ricinus communis* and *Vernicia montana*. This modified method will accelerate functional studies of tung tree at the molecular level and also shed light on plants lacking a transgenic system.

## Data Availability

Not applicable.

## Supplementary Materials

The following supporting information can be downloaded at: https://www.mdpi.com/article/10.3390/plants11101315/s1. Additional supporting information can be found in the online version of this article. Table S1: The primers of mCherry genes used for PCR amplification based on genomic DNA and qRT-PCR amplification based on cDNA.
